# Editorial: Safety in Collaborative Robotics and Autonomous Systems

**DOI:** 10.3389/frobt.2022.949214

**Published:** 2022-07-01

**Authors:** Ashwin Dani, Zhen Kan, Rushikesh Kamalapurkar, Nicholas Gans

**Affiliations:** ^1^ Electrical and Computer Engineering, University of Connecticut, Mansfield, CT, United States; ^2^ Department of Automation, University of Science and Technology of China, Hefei, China; ^3^ Mechanical and Aerospace Engineering, Oklahoma State University, Stillwater, OK, United States; ^4^ University of Texas at Arlington Research Institute, Arlington, TX, United States

**Keywords:** safe control, Control Barrier Functions (CBFs), digital twins, safe human robot interaction, Safe reinforcement learning

Safety is one of the most crucial aspects in collaborative robotics and autonomous systems. Recent developments in this field include advances in sensing, perception, and control, such as ways of presenting information using AR/VR, machine learning for control, and AI technologies. Developments of safety in human-robot collaboration can be applied to a variety of contexts, including industrial manufacturing, biomedical and surveillance applications.

The goal of this Research Topic is to collect high-quality articles in the areas of sensing, learning, planning, and control design to achieve or improve upon the current practice of *safety in collaborative robotics and autonomous systems*. Eight original research articles are collected from 34 authors, including theory and applications focusing on the developments in three major areas, namely, safety in human-robot collaboration, safety in planning and control, and safety in multi-robot collaboration. Detailed explanation of the contributions of each of the research articles is given below.

## Safety in Human-Robot Collaboration

Research article Sinha and Wang develops a novel deep reinforcement learning (RL) framework for task-constrained path generation problems of robotic manipulators from human demonstrated trajectories. Koopman operator theory is used to build a human intent model, which is then used in the reward function that can be used with a generic RL algorithm.

Research article Shi et al. focuses on safe, image-based visual servo control (IBVS) for human-robot interaction (HRI) applications, where the goal is to avoid collision with the human hand. The system described in the paper can avoid collision with human hands by learning the repulsive force using a Bayesian Deep Neural Network (DNN). The paper presents a learning-based method for estimating the repulsive field for obstacle avoidance tasks.

Research article Isaly et al. develops a safe combined motor and FES control system with the objective of maintaining the cadence of the rider near a target point for stationary motorized cycling assisted by functional electrical stimulation (FES) application, which is a popular therapy for people with movement impairments. Maximizing volitional contributions from the rider of the cycle can lead to long-term benefits such as increased muscular strength and cardiovascular endurance. Safety-ensured barrier functions are used to guarantee that cadence remains within a user-defined safe range, while minimal assistance is provided within the range to maximize effort by the rider.

Research article Ciullo et al. presents a supernumerary robotic limb (SRL) system that can help reduce the most common cause of work-related musculoskeletal disorders (WMSD), such as joint overloading, bad postures, and vibrations. SRLs are additional robotic body parts (e.g., fingers, legs, and arms) that can be worn by the workers, augmenting their natural ability, and reducing the risks of injuries. The authors design the SRL system with the aim to reduce the vibration transmitted along the arms and minimize the load on the upper limb joints. An off-the-shelf wearable gravity compensation system is integrated with a soft robotic hand and a custom damping wrist, designed starting from theoretical considerations on a mass-spring-damper model.

## Safety in Planning and Control

The objective of research article Mahmud et al. is to “develop safe reinforcement learning methods for deterministic nonlinear systems, with parametric uncertainties in the model, to learn approximate constrained optimal policies without relying on stringent excitation conditions. To that end, a model-based reinforcement learning technique that utilizes a novel filtered concurrent learning method, along with a barrier transformation, is developed in this paper to realize simultaneous learning of unknown model parameters and approximate optimal state-constrained control policies for safety-critical systems.”

Research article Davoodi et al. leverages probabilistic movement primitives (ProMPs), control Lyapunov functions (CLFs), and control barrier functions (CBFs) to create a novel means of teaching control by demonstrations. Human users perform a robot task multiple times, defining a distribution of acceptable trajectories, represented by a ProMP. CBFs and CLFs are automatically defined based on the distribution such that the robot trajectories stay relatively close to center but can deviate to optimize power consumption or avoid obstacles and can never leave the distribution into unknown regions.

## Safety in Multi-Robot Collaboration

In research article Dan et al., the authors formulate a novel object search/surveillance problem “wherein not only the persistent coverage, safety certificates and energy persistency but also task switches between search and surveillance are integrated.” The problem is solved using a novel constraint-based controller that can maintain a prescribed performance level.

Research article Douthwaite et al. discusses digital twins. Digital twins “allow critical ‘what if?’ and ‘why?’ questions to be answered within the design cycle, and the exploration of system choices and their impact on operability and deployment.” In this paper, the authors develop a modular digital twinning framework that allows them to “simulated and real-world data to assess the safety of collaborative robotic processes both during the design phase and during operation.”

In conclusion, the research articles in the Research Topic present advances in the state-of-the-art of learning, sensing, and control for safety of multi-robot and human-robot interaction/collaboration.

